# Genotyping-by-sequencing-based QTL mapping reveals novel loci for *Pepper yellow leaf curl virus* (PepYLCV) resistance in *Capsicum annuum*

**DOI:** 10.1371/journal.pone.0264026

**Published:** 2022-02-17

**Authors:** Muhammad Irfan Siddique, Joung-Ho Lee, Jung-Hwan Ahn, Meirina Kartika Kusumawardhani, Ramadhani Safitri, Asep Harpenas, Jin-Kyung Kwon, Byoung-Cheorl Kang

**Affiliations:** 1 Department of Agriculture, Forestry and Bioresources, Research Institute of Agriculture and Life Sciences, Plant Genomics and Breeding Institute, College of Agriculture and Life Sciences, Seoul National University, Seoul, Korea; 2 ECOSEED, Gimje-si, South Korea; 3 Department of Plant Pathology, East West Seed Indonesia, West Java, Indonesia; KGUT: Graduate University of Advanced Technology, ISLAMIC REPUBLIC OF IRAN

## Abstract

Disease caused by *Pepper yellow leaf curl virus* (PepYLCV) is one of the greatest threats to pepper (*Capsicum* spp.) cultivation in the tropics and subtropics. Resistance to PepYLCV was previously identified in a few *Capsicum* accessions, but no resistance QTLs have been mapped. This study aimed to elucidate the genetics of PepYLCV resistance in *C*. *annuum* L. Augmented inoculation by the viruliferous whitefly *Bemisia tabaci* was used to evaluate parental lines and an F_2_ segregating population derived from a cross between resistant *C*. *annuum* line LP97 and susceptible *C*. *annuum* line ECW30R. Final evaluation was performed six weeks after inoculation using a standardized 5-point scale (0 = no symptoms to 4 = very severe symptoms). A high-density linkage map was constructed using genotyping-by-sequencing (GBS) to identify single-nucleotide polymorphism (SNP) markers associated with PepYLCV resistance in the F_2_ population. QTL analysis revealed three QTLs, *peplcv-1*, *peplcv-7*, and *peplcv-12*, on chromosomes P1, P7, and P12, respectively. Candidate genes associated with PepYLCV resistance in the QTL regions were inferred. In addition, single markers Chr7-LCV-7 and Chr12-LCV-12 derived from the QTLs were developed and validated in another F_2_ population and in commercial varieties. This work thus provides not only information for mapping PepYLCV resistance loci in pepper but also forms the basis for future molecular analysis of genes involved in PepYLCV resistance.

## Introduction

Begomoviruses cause severe diseases in major vegetable crops, especially in the tropics and subtropics of Asia and America. The genus Begomovirus is the largest in the family Geminiviridae and contains more than 200 species. Begomoviruses have been reported to contain monopartite and bipartite genomes [1, 2]. Recently, the two begomoviruses *Tomato yellow leaf curl virus* (TYLCV) and *African cassava mosaic virus* (ACMV) were listed among the 10 most important plant viruses according to the economic losses they cause and their scientific importance [3, 4].

The begomovirus *Pepper yellow leaf curl virus* (PepYLCV) is a destructive pathogen that severely limits pepper production in South Asian countries. PepYLCV consists of a circular ssDNA of about 2.75 kb and is mainly transmitted via the whitefly insect vector *Bemisia tabaci* [5, 6]. Upon PepYLCV infection, pepper plants show cupping, the deformation of leaves and stunted plant growth, and a reduction in fruit size. Following severe infection, plants show inhibited pollen development and the dropping of flower buds, which leads to the absence of fruit set [7]. These symptoms complicate the adoption of appropriate preventative measures at early stages [4, 8]. Despite its importance, the occurrence of begomoviruses as a major pathogen of pepper is relatively recent compared with that of tomato [4]. For instance, among the five begomoviruses that cause leaf curl disease in pepper in the Americas, only one was identified in Asian countries early in the year 2000 [9]. Since then, the number of begomoviruses that infect pepper has increased dramatically in Asian regions of pepper cultivation, including Bangladesh, China, India, Indonesia and Pakistan, and about 29 species have been reported, with a vast diversity of virus strains [4, 8].

Current begomovirus management approaches depend mainly on insecticides to control the whitefly vector. However, such strategies are ineffective when whiteflies transmit the virus to other plants before disease symptoms become apparent in the field or greenhouses. Moreover, some vector insects have become resistant to specific insecticides [3, 10]. An alternative method of begomovirus management is to develop resistant cultivars. Indeed, begomoviruses resistance has been identified in accessions of several *Capsicum* species, such as *C*. *chinense* BG-3821, a Mexican line [11]. Resistance has also been reported in *C*. *annuum*, including EC-497636, GKC-29, BS-35 [12], Kalyanpur Chanchal [13] and breeding lines from the World Vegetable Center collection [4, 8, 14].

Screening for pepper resistance against begomoviruses is challenging because the sap inoculation method has not been successful to date [2, 4, 7]. Moreover, the presence of multiple pathogens can hinder the evaluation of resistance under conditions of natural disease incidence in the field. Screening via grafting and the micro-cage technique has also been reported, but these can be time-consuming and labor-intensive [7, 12]. The agroinfiltration method has been used to evaluate resistance using PepYLCV Indonesia strains [2, 15–17]. However, this method requires sophisticated cloning and transformation to inoculate the plants. The augmented inoculation of plants by the viruliferous whitefly *B*. *tabaci* has been used for reliable screening to evaluate resistance to YLCV in pepper and tomato [4, 8, 18, 19].

Inheritance studies are required to identify novel sources of resistance to PepLCV and to develop closely linked markers that facilitate introgression of PepYLCV resistance into commercial pepper varieties. Genetic analyses of resistance to begomoviruses has revealed complex modes of inheritance, including polygenic, monogenic and strain-specific types of resistance [7, 20–23]. For instance, some resistance against PepYLCV is governed by monogenic recessive genes with additive, dominant and epistatic effects [21, 24]. By contrast, mapping of a PepYLCV resistance locus using single sequence repeat (SSR) markers in an F_2_ segregating population revealed a single dominant resistance gene on pepper chromosome 6 [18]. This locus was delimited to within 15.7 cM, between two adjacent SSR markers CA516044 and PAU-LC-343-1, at genetic distances of 6.8 cM and 8.9 cM, respectively. The discrepancies concerning the modes of resistance inheritance may be due to the use of different regional virus strains and inoculation methods; for example, the use of open-field tests versus artificial inoculation conditions based on viruliferous whitefly as a vector [25]. Furthermore, virus strains are continuously evolving from monopartite to bipartite species, which may overcome the previous resistance.

In this study, we report genetic mapping of novel resistance genes to PepYLCV Indonesia strain using genotyping-by-sequencing-based QTL mapping in an F_2_ segregating population of pepper. Markers linked to the identified QTLs were developed and validated using several resistant pepper varieties.

## Materials and methods

### Plant materials

The resistant *C*. *annuum* line LP97 and the susceptible *C*. *annuum* line ECW30R were provided by EcoSeed (Gimjae, Korea) (Table 1). The F_1_ plants derived from a cross between the two lines were self-pollinated to develop a segregating F_2_ population named LP97-F_2_. The 150 LP97-F_2_ seedlings, 13 resistant (LP97) and 13 susceptible (ECW30R) control plants (Table 1) were grown in the greenhouse of East West Seeds Indonesia (EWINDO). An additional F_2_ population derived from the resistant commercial hybrid “Eagle F_1_” named Eagle-F_2_ was also screened and used for marker validation (Table 1). In this screening trial, the 184 Eagle-F_2_ seedlings with 40 resistant (Eagle F_1_) and 40 susceptible (ECW30R) control plants were evaluated for PepYLCV resistance as described above. Additional resistant commercial F_1_ hybrids were also used for marker validation (Table 1).

**Table 1:** Plant material used for mapping and marker testing.

No.	Cultivar	Type	Expected phenotype	Source
1	LP97	Double haploid	Resistant	Eco Seeds
2	ECW30R	Accession	Susceptible	Eco Seeds
3	LP97-F2	F_2_	Segregating population	Eco Seeds
4	Eagle-F2	F_2_	Segregating population	Eco Seeds
5	Eagle-F1	Commercial F_1_	Resistant	Eco Seeds
6	SONAL-F1	Commercial F_1_	Resistant	Eco Seeds
7	Sarangi-F1	Commercial F_1_	Resistant	Eco Seeds
8	Vikrant-F1	Commercial F_1_	Resistant	Eco Seeds
9	Armour-F1	Commercial F_1_	Resistant	Eco Seeds
10	Romyz1 F1	Commercial F_1_	Resistant	Eco Seeds

### Resistance screening

The PepYLCV Indonesia strain was used to screen the F_2_ segregating population and additional commercial F_1_ hybrids. The strain was maintained on susceptible infected pepper plants in a screen house of EWINDO. The seedlings were transferred to the screen house for resistance evaluation using viruliferous whitefly as a vector [4, 18]. To maintain the whitefly population, highly susceptible *Solanum lycopersicum* (local cultivar: Tombatu) and *S*. *melongena* (local cultivar: Mustang) plants were placed 90 cm apart among the test plants. A screen house was formed from insect-proof nylon net to prevent whitefly escape and entry by other insects. The plants were assessed according to disease severity grades 0 to 4 (S1 Fig) as described previously [4] with slight modification. The final evaluation for resistance and susceptibility was carried out 6 weeks after the transfer of seedlings to the screen house.

### Preparation of GBS libraries and SNP identification

Genomic DNA was extracted from young leaf tissues of plants from the F_2_ segregating population at the seedling stage using the hexadecyl trimethyl ammonium bromide (CTAB) method described by [26]. Genotyping-by-sequencing was performed as described previously [27, 28]. Briefly, genomic DNA of F_2_ and control plants was diluted, and the concentration was adjusted to 20 ng μL^−1^. The DNA was digested with *Eco*RI and *Mse*I; after ligation of adaptors to the digested DNA, the libraries were amplified with selective primers. The amplified libraries consisting of 92 F_2_ samples and two replicates of susceptible (ECW30R) and resistant (LP97) parents were pooled into a single tube. The pooled libraries were sequenced using an Illumina HiSeq2000 at Macrogen (Macrogen, Inc., Seoul, Korea). Trimming and quality control of the GBS raw data were performed using CLC Genomics Workbench v6.5 (Qiagen, Aarhus, Denmark) with a minimum read length of 80 bp and a minimum quality score of Q20. Filtered reads were aligned to the *C*. *annuum* cv. Dempsey reference genome [Unpublished] using the Burrows-Wheeler Aligner (BWA). Filtering and SNP calling were performed using the Genome Analysis Toolkit (GATK) Unifed Genotyper version 3.3–0. The SNPs in the F_2_ population were filtered with QUAL value >20 and a minimum read depth of three.

### Bin map construction, linkage analysis and QTL mapping

The SNPs that showed distorted and uneven segregation and more than 50% missing data were removed before linkage-map construction. To construct a linkage map, linkage bins were treated as genetic markers. The sliding window approach was used to impute the missing data and identify recombination break points as described previously [27]. To assign genetic positions to the bins, arranged bins were mapped with a LOD (logarithm of the odds) threshold of 3.0 and a distance threshold of 30 cM using CarthaGene software. The Kosambi mapping function was used to convert genetic distances between markers. CIRCOS 0.66 software v0.66 was used to compare the collinearity of the bin locations between the physical position and the genetic position [29]. QTL analysis was performed using the composite interval mapping using Windows QTL Cartographer 2.5 [30]. The 1,000-permutation test (*P* < 0.05) was performed to determine the LOD threshold for the significance of each QTL. Explanations of the phenotypic variance (PV) and additive effects for each QTL were also obtained using this software. In addition, R/qtl was used to verify the QTL results (https://cran.r-project.org/web/packages/qtl).

### Candidate gene and intergenic SNP analysis

The physical positions of the QTLs in the pepper genome were marked using the genetic distance information of the corresponding bins. Candidate genes within the QTL regions were retrieved using the annotated genes from the *C*. *annuum* cv. Dempsey reference genome [Unpublished]. Functions for candidate genes were annotated using BLAST2GO version 5 [31]. To annotate the intra- and intergenic variants in the QTL regions, snpEff version 4.3t [32] was used and the SNP information in the QTL regions was classified using the results generated by snpEff.

### Marker development and validation

The flanking sequences of the linked SNPs in the QTL regions were retrieved from the *C*. *annuum* cv. “Dempsey” reference genome database [Unpublished] and the online OligoAnalyzer tool (IDT International, Iowa, USA) was used to design primers. All DNA sequences containing SNPs were subjected to PCR amplification, and polymorphisms were confirmed between the resistant and susceptible parents by Sanger sequencing. In the next step, high-resolution melt (HRM) analysis was performed to validate the SNP markers. The HRM analysis was performed as described by [33].

## Results

### Phenotypic evaluation of parental lines and the mapping population

**Fig 1:**
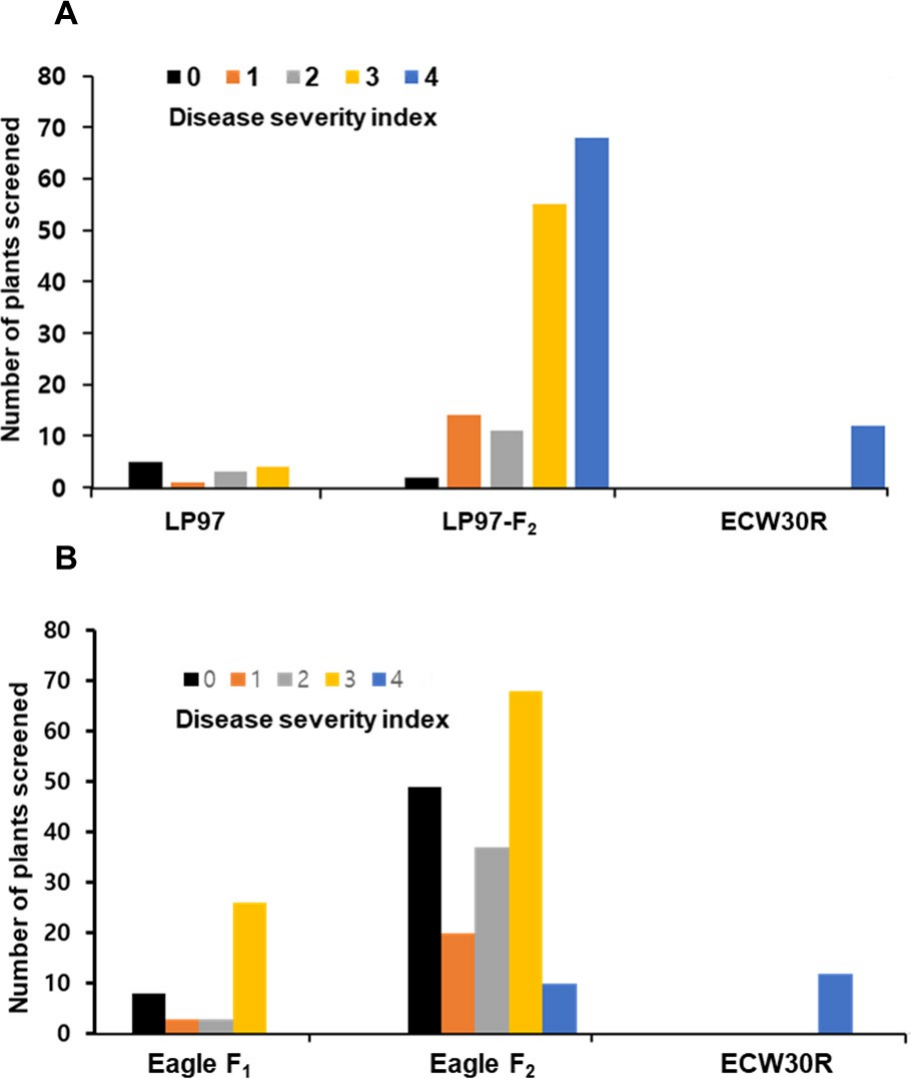
Frequency distribution of disease severity. Evaluation of resistance of the LP97-F_2_ population and control plants (A) and of Eagle-F_1_ and Eagle-F_2_ plants (B) to *Pepper yellow leaf curl virus* (PepYLCV) Indonesia strain performed 6 weeks after transfer of seedlings to the screen house. The disease severity index from 0 to 4 was used.

The resistance of the F_2_ plants to PepYLCV was evaluated following inoculation by viruliferous whiteflies (*B*. *tabaci*). The plants were assessed according to disease severity grades 0 to 4 from June to September 2018 (S1 Fig). Thirty-day-old seedlings were moved to the screen house and final evaluations were carried out six weeks after the plants were transferred to the screen house. Out of 150 F_2_ plants, 27 plants were assessed between grades 0 to 2, and 123 plants were scored as 3 or 4 (Fig 1A). Among 13 plants of the resistant control LP97, 9 were scored between grades 0 to 2, and 4 plants were scored at grade 3, however, no plant showed complete susceptibility (Figs 1A and S2). All 13 plants of the susceptible control line ECW30R were scored at grade 4 at the final evaluation (Figs 1A and S2). The frequency distribution curve showed a negatively skewed distribution, indicating the involvement of QTLs in controlling resistance (Fig 1A).

An additional F_2_ population, “Eagle-F_2_”, which contained 184 individuals derived from the commercial resistant F_1_ hybrid “Eagle-F_1_” was also evaluated under the same screen house conditions in 2019 from March to June. Out of 184 F_2_ plants, 69 plants were scored at grades 0 or 1 and 115 were scored between grades 2 and 4 (Figs 1B and S2). For marker validation, F_2_ plants scored at grades 0 or 1 were considered to be resistant “R” and plants between grades 2 to 4 were designated as susceptible “S”. Eagle-F_1_ and ECW30R were used as resistant and susceptible controls, respectively. Out of 40 Eagle-F_1_ plants, 11 were scored at grades 0 or 1 and 29 plants were scored at grades 2 or 3 (Figs 1B and S2). However, no plant was scored at grade 4, whereas all susceptible control “ECW30R” plants were scored at grade 4 (Fig 1B). These results also indicated that the resistance against PepYLCV is governed by multiple loci.

### SNP discovery and linkage-map construction

**Fig 2:**
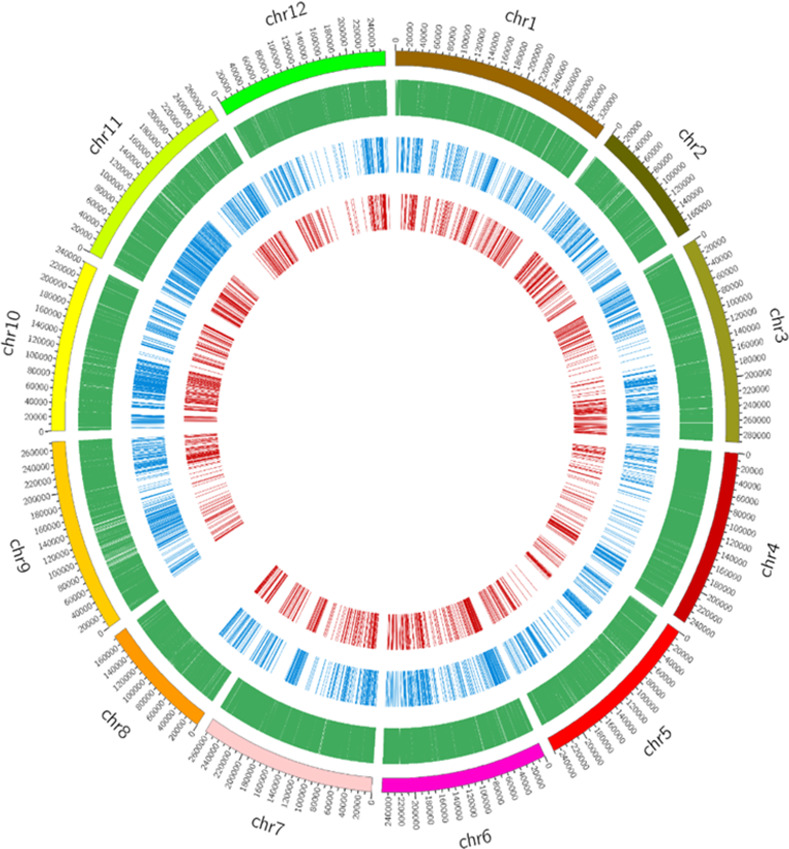
Genome-wide distribution of SNPs and bins across pepper genomes. The outermost box with scale represents the 12 pepper chromosomes. The green histogram represents the density of raw SNPs; the blue histogram indicates the SNPs that are polymorphic between LP97 and ECW30R that were used for bin construction; the red histogram indicates the bins of adjacent SNPs that were used for linkage-map construction.

We performed genotyping-by-sequencing to obtain the single nucleotide polymorphism (SNPs) markers to construct a genetic linkage map of LP97-F_2_ population. The genotyping of 92 randomly selected F_2_ samples and two replicates for each control was performed using genotyping-by-sequencing (GBS) following digestion with *Eco*RI and *Mse*I. The Illumina paired-end sequencing of the GBS libraries and controls generated 247.8 million raw reads. After the trimming of raw reads with quality filters and alignment of the reads to the reference genome, 55,460 SNPs were obtained (Fig 2). The SNP density distribution revealed that the SNPs were uniformly distributed across the chromosomes (Fig 2). In the next step, after parental calling between resistant and susceptible controls, the removal of more than 50% of missing data and filtering unequally distributed SNPs, a total of 3,249 high-quality SNPs were obtained, which were used for the construction of bins (Fig 2).

**Fig 3:**
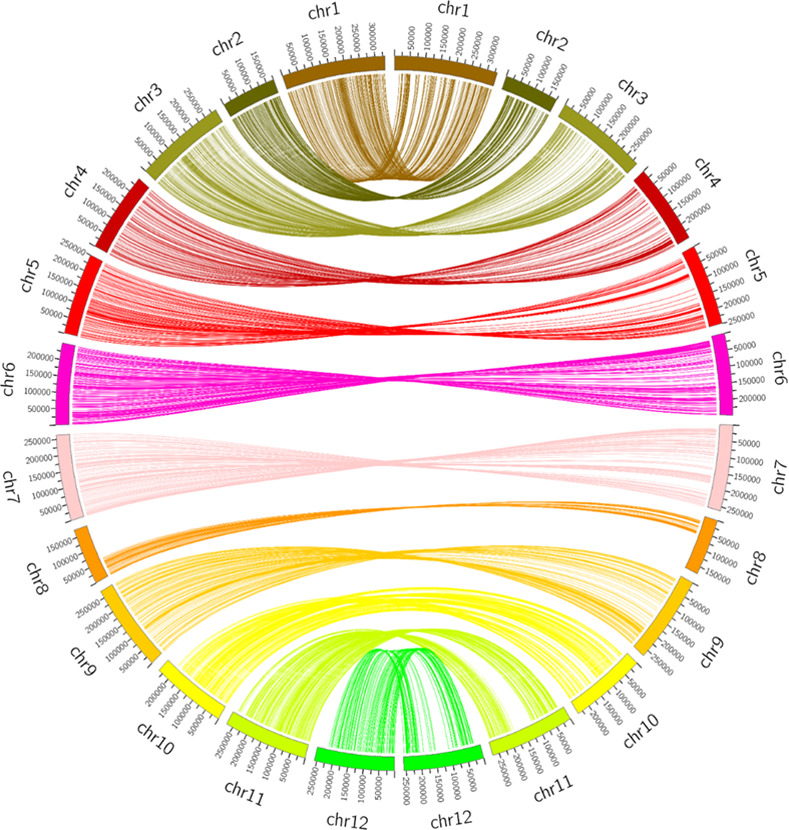
Collinearity between the genetic map and the physical map of pepper. The outer circle represents the total number of chromosomes (chr = ID) on left side and linkage groups (chr = ID) on right side; markers located within linkage groups are linked to the parallel position on chromosomes by different colored lines in the inner circle.

The sliding window method was employed to estimate the genotyping error and missing data before the construction of a genetic linkage map [27, 28]. First, recombination breakpoints were ascertained by sliding 15 SNPs sequentially as a single window, which resulted in 1,140 bins (Fig 2 and Table 2). A recombination bin map of the F_2_ population showed the admixture of resistant, susceptible and heterozygote segments (S3 Fig). A high-density genetic linkage map was then constructed using 3,249 high-quality SNPs, which yielded 1,140 bins representing a total genetic distance of 1,737.1 cM in length (Table 2). Among the 12 linkage groups, maximum and minimum genetic distances of 214.9 and 72.9 cM were obtained for chromosomes P1 and P8, respectively (Table 2). To assess the quality of the genetic map, collinearity analysis was conducted to compare the physical and genetic positions of the bin (Fig 3). Most of the bins showed the same order on the corresponding chromosomes of the reference genome, except for P10 and P12, which deviated slightly in the collinearity analysis (Fig 3). This high-density genetic linkage map was used in further QTL analysis for PepYLCV resistance.

**Table 2:** Summary of the bins and genetic linkage map for the LP97-F_2_ population.

Chromosome	Number of SNPs	Number of bins	Physical length of bin (Mb)	Genetic distance of Bin (cM)
P1	269	125	332.7	214.9
P2	233	91	175.7	128.4
P3	273	117	291.1	154.2
P4	141	60	248.3	90.6
P5	221	82	250.1	120.8
P6	274	116	249.4	183.6
P7	238	93	262.8	147
P8	84	39	173.4	72.9
P9	388	85	271.6	123.6
P10	396	124	240.1	186.7
P11	550	139	271.1	193.2
P12	182	69	257.6	121.2
Total	3,249	1,140	3,023.90	1,737.10

### Identification of QTLs for resistance to PepYLCV

**Fig 4:**
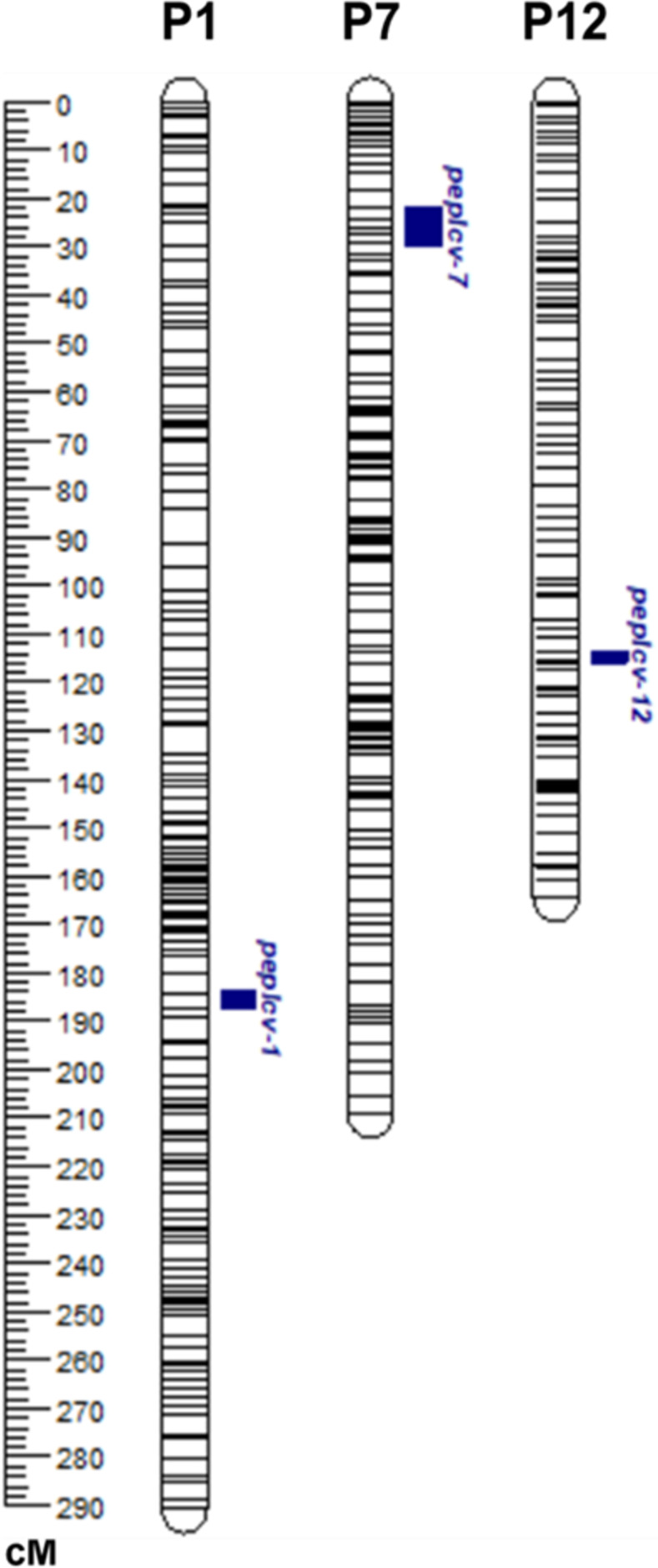
A bin based linkage map showing the locations of QTLs for resistance to PepYLCV. The genetic distance is shown in centimorgans (cM). LP97-F_2_ plants were evaluated for resistance to PepYLCV Indonesia strain in a screen house.

The genetic map of the LP92-F_2_ population was used to detect the QTLs for resistance to PepYLCV. In total, three QTLs for PepYLCV resistance that explained phenotypic variation (R^2^) ranging from 6.3 to 31.7% were identified across the pepper genome (Fig 4 and Table 3). These QTLs for PepYLCV resistance were detected on chromosomes P1, P7 and P12 (Fig 4 and Table 3). The major QTL was on P7 at 21.01 cM, corresponding to 26–32 Mb in the reference genome, and was named *peplcv-7*. The QTL *peplcv-7* explained 31.7% of the phenotypic variation (R^2^) with a LOD score of 8.6 (Fig 4). Two minor QTLs, *peplcv-1* and *peplcv-12*, were detected at 185.41 cM and 113.51 cM, corresponding to 254–256 Mb and 252–256 Mb on P1 and P12, respectively. The *peplcv-1* and *peplcv-12* QTLs explained 9.9% and 6.3% of the phenotypic variation (R^2^), respectively, with LOD scores of 2.7 and 4.2, respectively (Fig 4 and Table 3). The QTL results were verified using an alternative software R/qtl, which also detected similar QTLs at the same locations (S4 Fig). These QTLs were used for further investigation to develop single markers and predict candidate genes linked to PepYLCV resistance.

**Table 3:** QTL analysis for resistance to PepYLCV Indonesia strain in the LP97-F_2_ population using composite interval mapping.

QTLs	Chromosome	Genomic position (cM)	Flanking bins	Physical position (Mbp)	LOD	*R*^*2*^ *(%)*	Dominant effect
*peplcv-1*	1	185.41	Chr1-LCV-102–103	254–256	2.7	9.9	6.483
*peplcv-7*	7	21.01	Chr7-LCV-16–21	26–32	8.6	31.7	3.799
*peplcv-12*	12	113.51	Chr12-LCV-94–96	252–256	4.2	6.3	10.936

### Potential candidate genes that confer resistance to PepYLCV

We searched for candidate genes related to PepYLCV resistance within the detected QTL regions. The flanking sequences of the QTL regions were retrieved from the *C*. *annuum* cv. “Dempsey” reference genome database [Unpublished] using upper and lower delimiting bin markers (Table 3). The QTL *peplcv-7* on P7 was inferred to contain 141 genes (S1 Table), including four genes that encode leucine-rich repeat domain-containing proteins, which are known to be associated with disease resistance (Table 4). By contrast, the QTL *peplcv-12* on P12 was inferred to contain 300 genes (S1 Table), including seven genes that encode leucine-rich repeat domain-containing proteins, two R1 gene for resistance to late blight, and one gene that encodes a protein with an Rx N-terminal domain, which is also known to be associated with disease resistance (Table 4). These genes represent potential candidate resistance genes for resistance to PepYLCV in pepper.

**Table 4:** Candidate genes associated with PepYLCV resistance and their functions.

Chromosome	Start position	End position	GO description
P7	31363005	31372454	Leucine-rich repeat N-terminal domain
P7	29255606	29258419	Leucine-rich repeat
P7	29241237	29245815	Leucine-rich repeat
P7	29162748	29164059	Leucine-rich repeat
P12	252998942	253002525	Late blight resistance protein R1
P12	253709733	253714975	Leucine-rich repeat
P12	253006918	253025995	Rx N-terminal domain
P12	255842999	255845707	Leucine-rich repeat
P12	253283936	253289693	Leucine-rich repeat N-terminal domain
P12	252990677	252994517	Late blight resistance protein R1
P12	255451163	255451900	Leucine-rich repeat
P12	253304077	253307441	Leucine-rich repeat N-terminal domain
P12	255851047	255854299	Leucine-rich repeat
P12	255367051	255373032	Leucine-rich repeat

### Validation of SNPs linked to PepYLCV resistance

**Fig 5:**
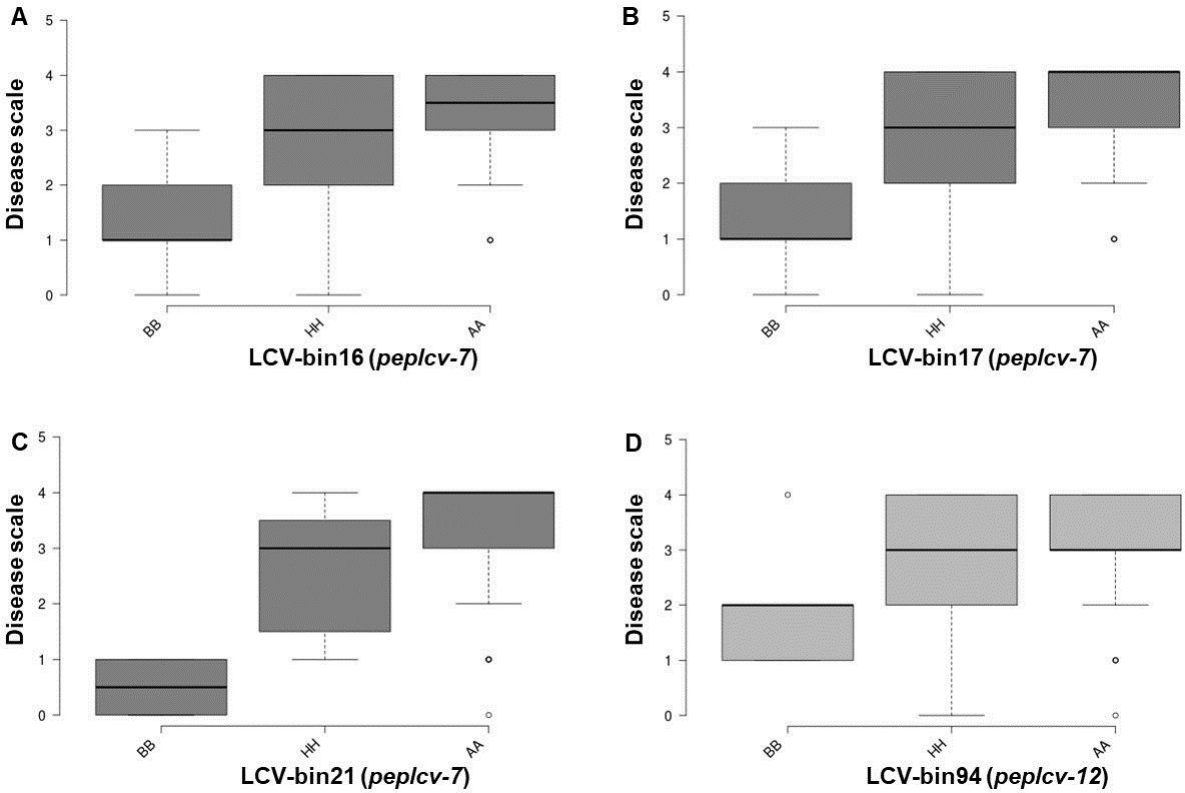
Box plots of tightly linked bins to QTLs from chromosome P7 and P12 of LP97-F_2_. A–C) LP97-F_2_ grouped based on the tightly linked bin to QTL *peplcv-7* against PepYLCV Indonesia strain. D) LP97-F_2_ grouped based on the tightly linked bin to QTL *peplcv-12* against PepYLCV Indonesia strain.

The single markers linked to the QTLs detected for resistance to PepYLCV were developed and validated. The flanking bin markers of QTL *peplcv-7* included the bins from Chr7-LCV-16 to Chr7-LCV-21 (Table 3). These bin markers were used for box-plots. For the bin marker Chr7-LCV-16, the level of PepYLCV resistance of the F_2_ plants that harbored the homozygous resistance allele was significantly higher than that for plants carrying the heterozygous and the homozygous alleles of the susceptible parents (Fig 5A). Similarly, a significant difference in the level of PepYLCV resistance was observed among F_2_ plants that carried a homozygous resistance allele for the Chr12-LCV-94 bin marker in the QTL *peplcv-12* (Fig 5B).

**Fig 6:**
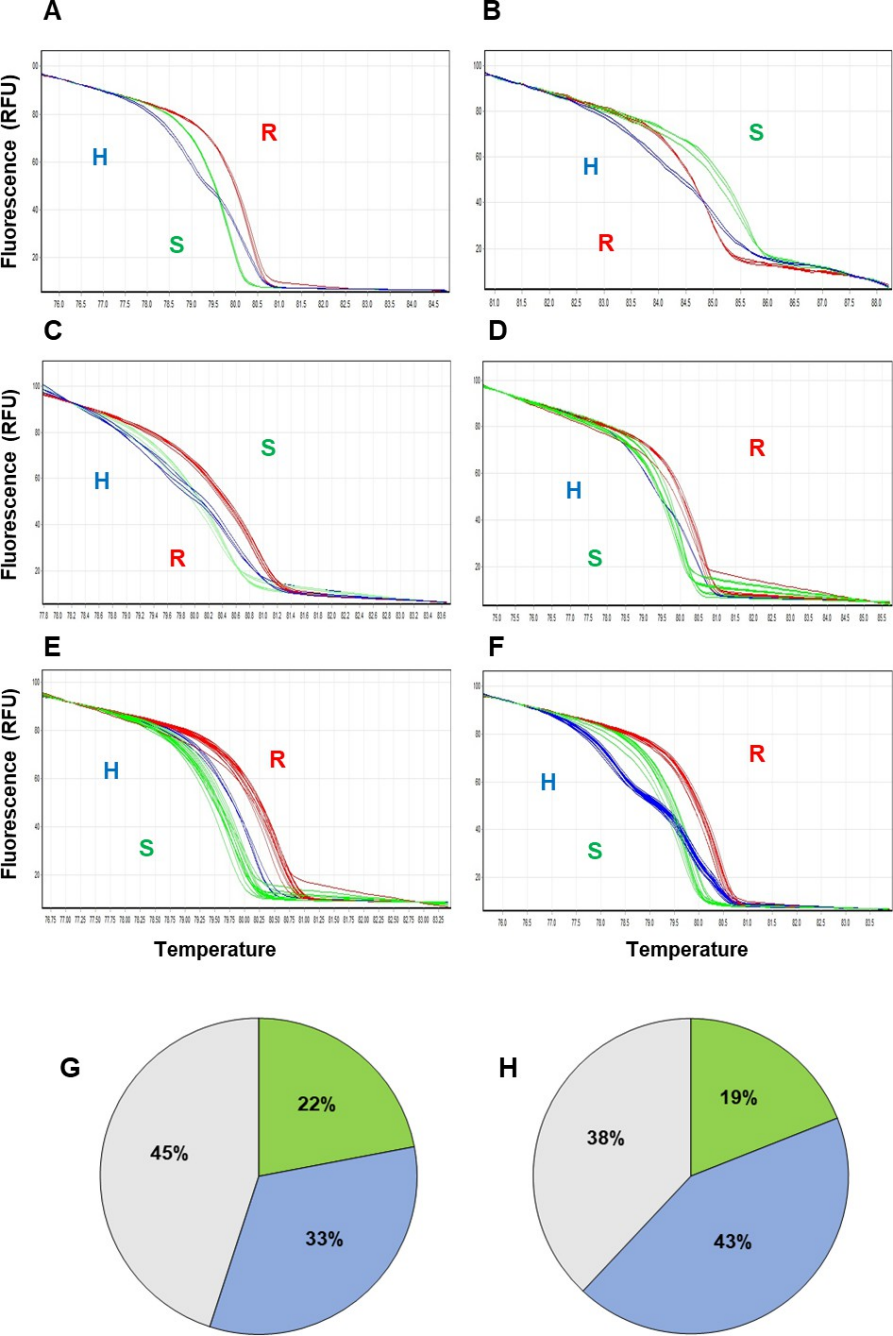
Validation of single markers. A) Parental survey with marker Chr7-LCV-7 on P7. B) Parental survey with marker Chr12-LCV-12 on P12. C) Marker Chr7-LCV-7 on P7 validation using resistant hybrids. D) Marker Chr12-LCV-12 on P12 validation using resistant hybrids. E) Marker Chr7-LCV-7 on P7 validation using additional F_2_ segregating population Egale-F_2_. F) Marker Chr12-LCV-12 on P12 validation using additional F_2_ segregating population Eagle-F_2_. G) Single marker Chr7-LCV-7 on P7 genotype–phenotype association in Eagle-F_2_. H) Single marker Chr12-LCV-12 on P12 genotype–phenotype association in the Eagle-F_2_ population. (G & H) Green, blue and grey represents exact phenotype-genotype matched, when heterozygous counted as susceptible and unmatched, respectively.

To validate these results further, the flanking sequences of SNPs in those bin markers were retrieved from the reference genome and additional markers were developed (Table 5). The high-resolution melt (HRM) genotyping assay was used to genotype the additional Eagle-F_2_ population and commercial F_1_ hybrids. The developed markers (Chr7-LCV-7 and Chr12-LCV-12) clearly distinguished the resistant and susceptible controls in a parental polymorphism survey (Fig 6A and 6B). The markers Chr7-LCV-7 and Chr12-LCV-12 were validated using commercial PepYLCV-resistant F_1_ hybrids (Table 1 and S2 Table). Among these, Sonal and Sarangi were determined to be resistance genotypes, whereas Eagle, Vikrant, Armour and Romyz1 were classified as heterozygous genotypes by the Chr7-LCV-7 marker on P7 (Fig 6C and 6D and S2 Table). The commercial F_1_ hybrids Eagle, Armour and Romyz1 were genotyped as resistant, whereas Sonal and Vikrant were determined to be the susceptible genotype for the Chr12-LCV-12 marker on P12 (Fig 6C and 6D and S2 Table).

**Table 5:** Marker sequence information used for QTL validation and genotyping.

Primer	Chromosome	Physical position (bp)	Sequence (5ʹ to 3ʹ)
Chr7-LCV-7_F	P7	26705882	CTGATAACTGACAGTTTAGATAGGAATTGG
Chr7-LCV-7_R	P7	26706033	CAACTCAGTCTATAACCGGTGTATG
Chr7-LCV-12_F	P12	256125396	TTTAATAAGTCGTGGAAGGACCGCA
Chr7-LCV-12_R	P12	256125557	CTATTAAAAGGACCGAGTTGGTTTGGC

The marker genotyping results for the Eagle-F_2_ population are shown in the (Fig 6E and 6F and S3 Table). The single marker Chr7-LCV-7 from the QTL *peplcv-7* showed a genotype and phenotype matching rate of 22% among F_2_ plants (Fig 6G and S3 Table). However, when the heterozygotic genotype was considered as susceptible, the marker genotype and phenotype matching rate increased to 55% (Fig 6G and S3 Table). Another single marker (Chr12-LCV-12) developed within QTL *peplcv-12* on P12 showed a genotype and phenotype matching rate of 20% among F_2_ plants (Fig 6H and S3 Table). Similarly, when the heterozygous genotype was considered as susceptible, the genotype and phenotype matching rate of the marker increased to 65% (Fig 6H and S3 Table). Furthermore, annotation of the intra- and intergenic variants in the QTL regions performed by snpEff revealed the presence of 366 intergenic SNPs, including 12 upstream gene variants and 8 downstream gene variants in QTL *peplcv-7* on P7 (S4 Table). By contrast, 102 intergenic SNPs, including 27 upstream variants and 43 downstream variants, were annotated in the QTL *peplcv-12* on P12 (S4 Table). These SNPs can be useful to develop molecular markers for PepYLCV resistance in pepper.

## Discussion

In this study, we mapped the novel QTL linked to PepYLCV resistance through viruliferous whitefly-mediated artificial screening of an F_2_ segregating population and using genotyping-by-sequencing based QTL mapping. To the best of our knowledge, this is the first report of QTLs associated with resistance to PepYLCV in pepper. We also developed and validated single markers linked to the resistant QTLs using a different F_2_ segregating population and commercial cultivars.

Previous reports described that resistance to PepYLCV is controlled by single recessive and dominant genes [17, 18, 34]. Genetic studies of virus resistance against leaf curl viruses during 1989–1990 showed that resistance was governed by monogenic recessive genes in pepper (*C*. *annuum*) [35]. Resistance to PepYLCV was evaluated using six genotypes that were resistant in the field. This revealed that PepYLCV resistance in ‘Punjab Lal’ pepper variety was recessive, because F_1_ plants were susceptible in artificially challenged conditions as well as in field conditions [20]. In a research report, germplasm that was resistant against *Chilli leaf curl virus* (ChiLCV-VNS; Varanasi isolate) and markers that were significantly linked to ChiLCV-VNS resistance were revealed that, resistance was governed by major recessive genes [21]. In another study which was carried out to elucidate the inheritance of resistance all types of gene actions including additive, dominant and epistatic gene interactions for virus resistance were revealed in interspecific crosses between *C*. *annuum* L. and *C*. *frutescens* L. [24]. An inheritance study of resistance to PepLCV in a partially compatible interspecific cross (*C*. *annuum* PBC-535 x *C*. *chinense* Bhut Jolokia) revealed the monogenic recessive nature of PepLCV resistance [7]. A recent study using the *C*. *annuum* BaPep-5 resistant accession inoculated with PepLCV by graft transmission and agroinfiltration showed that resistance to PepLCV was governed by a single recessive locus on chromosome P5, although susceptible and resistant plants did not segregate in a 3:1 ratio [17]. However, the evaluation of resistance against PepYLCV Indonesia strain mediated by whiteflies in the present study showed that resistance is controlled by QTL.

Tomato is a closely related species to pepper and TYLCV shares a high sequence similarity with PepYLCV [1, 2]. Several QTLs for resistance against TYLCV have been mapped in wild species of tomato, including *Solanum chilense* (*Ty-1*, *Ty-3*, *Ty-4*, and *Ty-6*), *S*. *habrochaites* (*Ty-2*), and *S*. *peruvianum* (*ty-5*) [3, 36–41]. Among these loci, *Ty-1* was initially mapped to chromosome 6 using a backcrossed population from a cross between *S*. *chilense* and *S*. *lycopersicum* [41]. *Ty-3* was mapped in *S*. *chilense* accessions (LA1932, LA2779, and LA1938) to chromosome 6 [37, 42]. Another resistance gene, *Ty*-*2*, was identified on chromosome 11 [39, 43] and was recently shown to encode a protein containing a nucleotide-binding domain and leucine-rich repeat (NB-LRR) [44]. A previous study on resistant gene analogues in pepper reported 5 different resistant gene classes including NB-LRR conferring resistance against multiple viruses such as *Cucumber mosaic virus* (CMV), *Chilli veinal mottle potyvirus* (ChiVMV), *Chilli leaf curl virus* (ChiLCV) [45]. Here, we identified a total of 11 (NB-LRR) genes as candidate resistant genes in the QTL regions. Among these, four were in QTL *peplcv-7* and seven were in QTL *peplcv-12* vicinity. In tomato, the TYLCV resistance locus *Ty-4* was mapped to chromosome 3 and a locus for recessive resistance, *ty-5*, was mapped to chromosome 4 and encodes a messenger RNA surveillance factor Pelota (*Pelo*) that is associated in the ribosome recycling-phase of protein synthesis [46]. Recently, a resistant locus *pepy-1* on pepper chromosome 5 was fine mapped in *C*. *annuum* and found to encode an RNA surveillance factor Pelota in BaPep-5 variety [17]. In tomato, a major begomovirus-resistant QTL, *Ty-6*, was mapped to chromosome 10 and strong resistance against TYLCV was obtained when *Ty-*6 was pooled with *Ty-3* or *ty-5* [3, 38]. Further synteny and collinearity analyses would identify any conserved QTL region between pepper and tomato that is responsible for PepYLCV resistance.

Molecular markers linked to potential resistance loci can provide information for early selection and are useful for breeding horticultural crops resistant to viruses [47]. Several molecular markers associated with virus resistance loci in pepper and tomato has been developed to expedite marker-assisted breeding [48, 49]. Introgression of resistance genes into cultivated varieties has been the principal route of breeding cultivars that are resistant to viruses such as *Tomato yellow leaf virus* (TYLCV), *Tomato spotted wilt virus* (TSWV), *Pepper yellow leaf curl virus* (PepYLCV) and *Chilli veinal mottle virus* (ChiVMV) [49, 50]. The major markers linked to tomato yellow leaf disease (TYLCD) that have been developed to date include a closely linked molecular marker, SCAR1, for screening of the *Ty-1* locus [51], P6-25 and FLUW25 for evaluation of *Ty-3*, *Ty-3a*, and *Ty-3b* loci [37, 50, 52], and SCAR2 and P1-16 for the detection of *Ty-2* [39]. Despite this progress, fewer markers for resistance to PepYLCV have been reported.

Genotyping-by-sequencing (GBS)-based SNP markers represent an advancement in marker-assisted selection. In this study, we developed and validated new markers based on GBS-SNPs, named Chr7-LCV-7 and Chr12-LCV-12, for the marker-assisted selection for PepYLCV resistance breeding in pepper. The single marker Chr7-LCV-7 developed for the QTL *peplcv-7* on P7 showed a genotype and phenotype matching rate in F_2_ plants of 22%. However, when the heterozygous genotype was considered as susceptible, the marker genotype and phenotype matching rate increased to 55%. Another single marker, Chr12-LCV-12, which was developed for the QTL *peplcv-12* on P12 showed a genotype and phenotype matching rate in F_2_ plants of 20%. However, when the heterozygous genotype was considered susceptible, the marker genotype and phenotype matching rate increased to 65%. These markers can therefore improve the accuracy of selection during PepYLCV-resistance breeding in pepper. In this study, the validation of markers using commercial resistant pepper genotypes revealed a correlation between the phenotypic and genotypic data. The commercial hybrids Sonal and Sarangi showed a resistant genotype with marker Chr7-LCV-7, whereas the marker Chr12-LCV-12 defined them as susceptible. This discrepancy might be because the resistance for PepYLCV from different sources has been incorporated in these commercial F_1_ hybrids.

## Conclusions

This study provides information regarding the genetic mapping of loci that confer resistance against PepYLCV. We reported the first PepYLCV-resistance QTLs and mapped them to pepper chromosomes P1, P7 and P12 using whitefly-mediated artificial screening and GBS-based linkage mapping approaches. The detected QTLs explained in total 47.9% of the phenotypic variation (R^2^) in a segregating F_2_ population. We further developed single markers linked to the resistance QTLs on P7 and P12 and validated them in an additional F_2_ population and commercial resistant F_1_ hybrids. The novel resistance loci and markers developed here will accelerate breeding programs for PepYLCV resistance in pepper.

## Supporting information

S1 Fig Disease scale used for resistance evaluation. (TIF)

S2 Fig Resistant and susceptible controls. (TIF)

S3 Fig Bin map of LP97-F_2_ population. (TIF)

S4 Fig QTLs verification using Rqtl and comparison of WinQTL Cartographer results. (TIF)

S1 Table Candidate genes in QTL regions associated with resistance to PepYLCV. (XLSX)

S2 Table Marker validation using resistant commercial F_1_ hybrids. (XLSX)

S3 Table Marker validation using an additional F_2_ population. (XLSX)

S4 Table Intergenic SNPs in QTL regions. (XLSX)
