# Supplementary figures and images for: Genotyping-by-sequencing-based QTL mapping reveals novel loci for Pepper yellow leaf curl virus (PepYLCV) resistance in Capsicum annuum
Source: PLoS One. 2022 Feb 17;17(2):e0264026. doi: 10.1371/journal.pone.0264026 (PMC8853517; doi:10.1371/journal.pone.0264026)

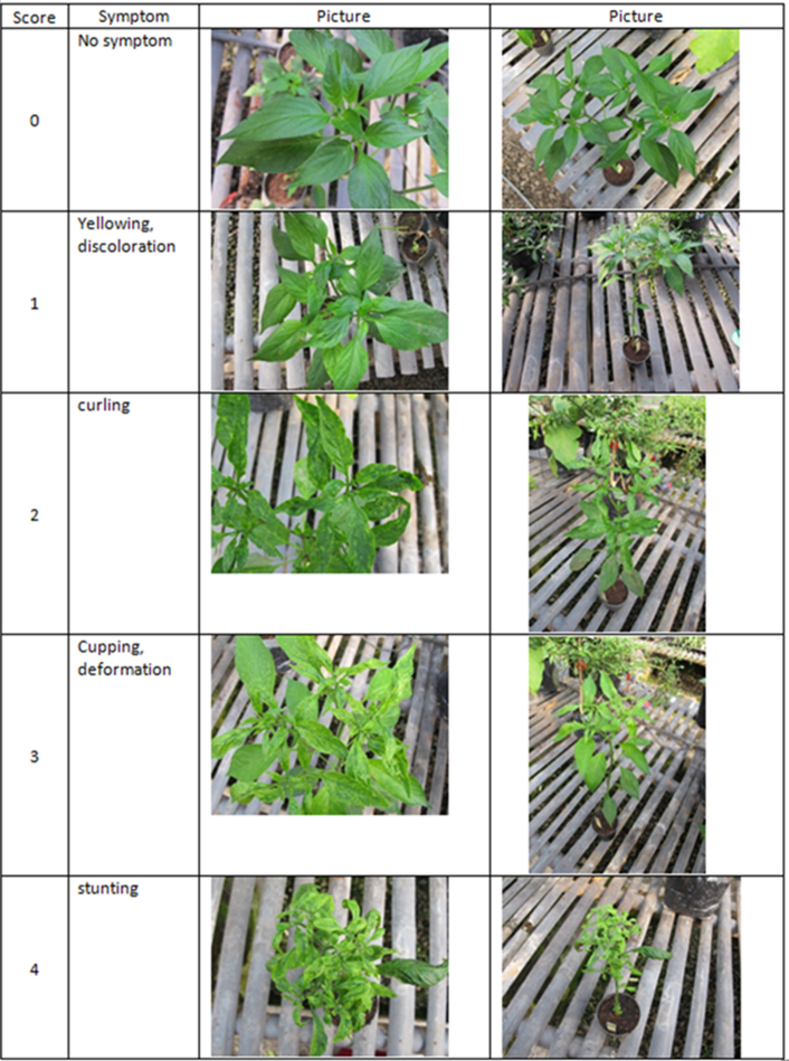

Supplement: S1 Fig — (TIF) [file pone.0264026.s001.tif]

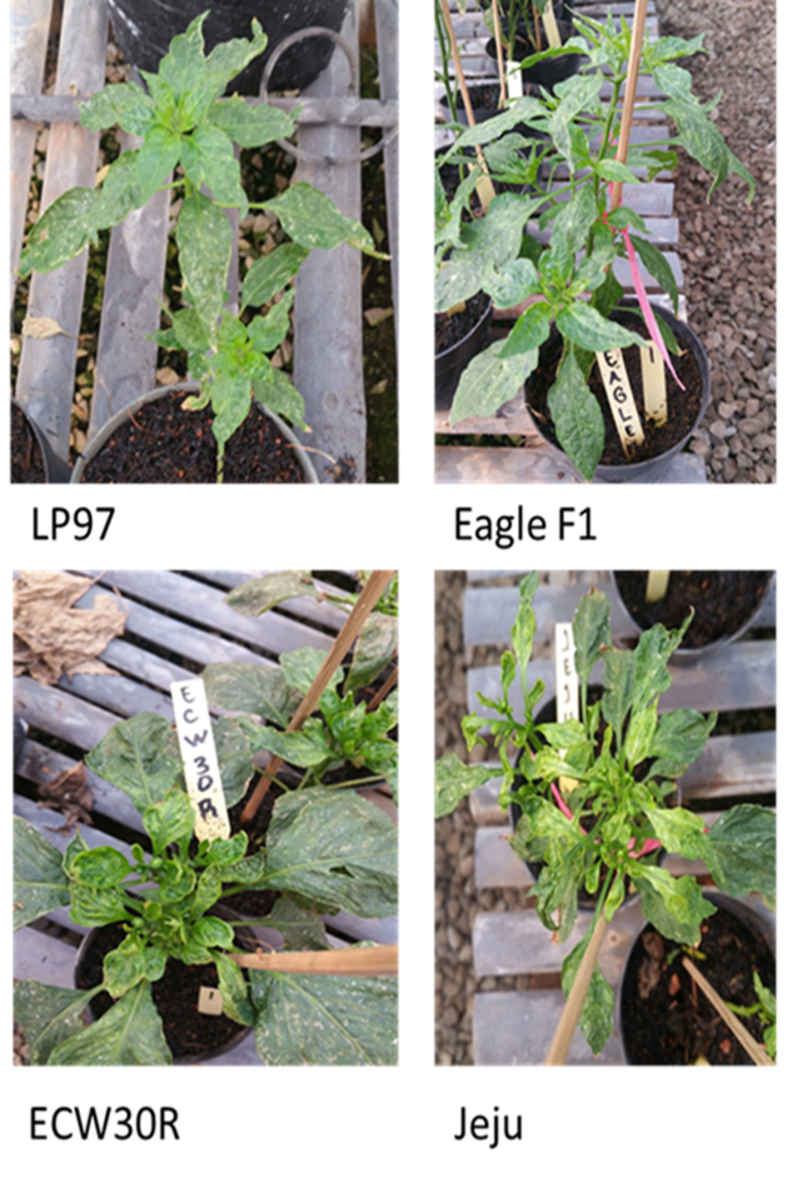

Supplement: S2 Fig — (TIF) [file pone.0264026.s002.tif]

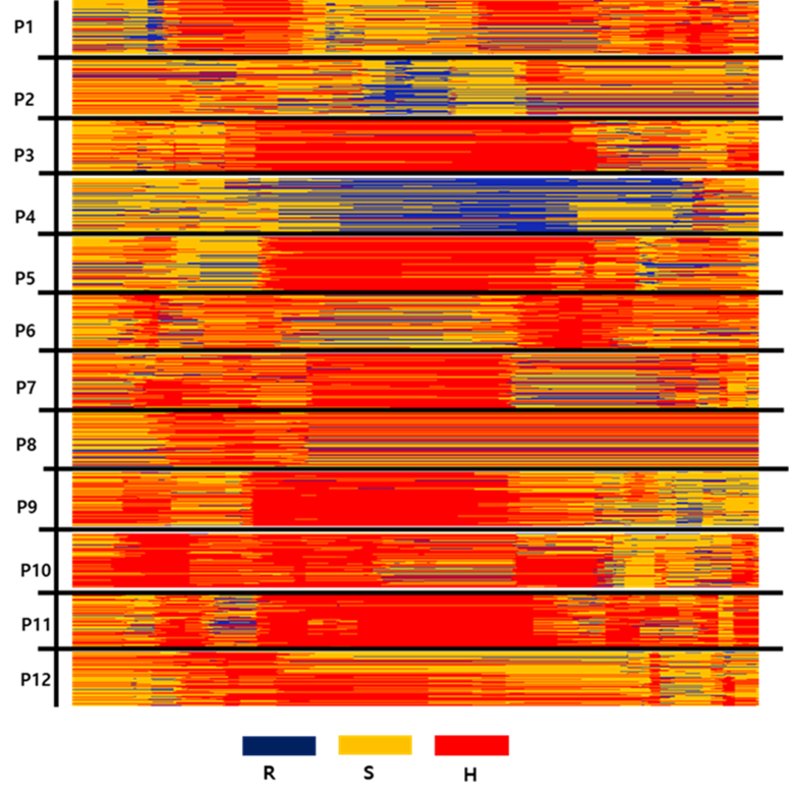

Supplement: S3 Fig — (TIF) [file pone.0264026.s003.tif]

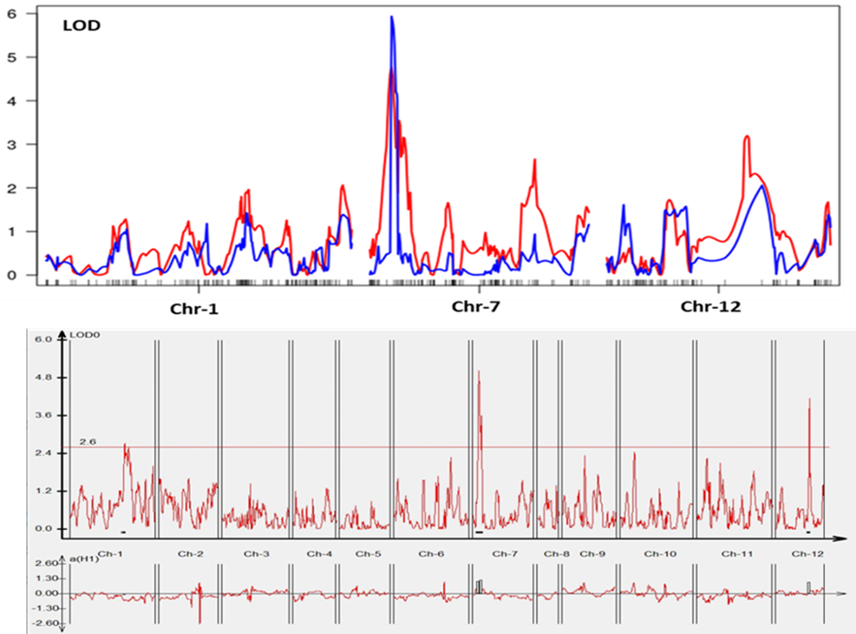

Supplement: S4 Fig — (TIF) [file pone.0264026.s004.tif]
